# Alveolar Abscess

**Published:** 1887-02

**Authors:** S. E. Knowles


### Alveolar Abscess—With Notes on Two Cases.

BY S. E. KNOWLES, M.D., D.D.S.

(Read before the California State Dental Association.)

The subject of alveolar abscess has by oft-repeated discussion been worn so nearly threadbare, that I am almost afraid the mere mention of my intention to try to engage your attention in its consideration will be sufficient to make you all weary at the start.

This subject, however often it has been under discussion, has not by any means been exhausted, as I hope to show you before the conclusion of this paper.

Type cases of this disease in the acute form are ordinarily easy of diagnosis, the swollen face, reddened skin and mucus membrane, loosened and perhaps decayed or discolored tooth, and tale of broken slumber, designating too plainly the source of the trouble. This acute form is almost always quite unmistakable, and careful examination will at once complete its identification. Other local disorders may be and sometimes are confounded with it, but when all the symptoms mentioned are present, it is a case of alveolar abscess, and no mistake.

Chronic alveolar abscess is, however, quite another affair. Its diagnosis may not only be difficult, but sometimes almost impossible, and if identified at all, perhaps by accident. Should the question " Are you familiar with the various forms of chronic alveolar abscess?" be put to each one of you, it may be presumed all answers would be in the affirmative, for it would be hard to expect any dentist to admit that there could be anything not perfectly well known about this disease.

Before presenting the account of the two cases referred to in the title of this paper, a hasty review of some of the anomalies of this form of abscess may prove to be of interest.

One anomaly is a case in which no fistulous opening exists, in which case the bone surrounding the seat of trouble becomes, so to speak, distended, the alveolar condition naturally existing in the alveolar process becoming exaggerated, and often causing the appearance of a tumor of considerable size. This, if occurring on a fragment of root left in the bone, in case of incomplete extraction, may be very difficult to diagnose, especially if the root be deeply seated and the gum completely healed over.

This form of disease is not very rare and has fallen under my observation four or five times. It may as well be here confessed that the first case seen was mistaken for an enormous hypertrophy of the cementum (exostosis), and great was my surprise when the tooth was extracted to find no enlargement of its hard tissues.

Another form is that in which a fistulous opening does exist but it is not apparent, and discharges at some point outside the mouth, and not upon the skin surface, as for instance into the nares or antrum.

I have never seen a case of discharge into the nares direct, but one is recorded by M. Fleischman, a writer in the British Medical Journal, and cited by Garretson, in which a girl of five years of age had a discharge of muco-purulent character for a period of three months, and which was eventually traced to a right superior canine tooth, the extraction of which cured the case in a few days.

This case was not recognized by the gentlemen who reported it as one of alveolar abscess, but as offering "a good illustration of reflected irritation."

Cases of evacuation of the discharge into the antrum are, however, comparatively common, and five or six have fallen to my lot to treat.

Garretson considers that the most prominent cause of disease in this cavity primarily is tooth lesions, and when we come to consider the relation which the palatine roots of the first and second superior molar teeth bear to the floor of the antrum, the disease of the one would, as a matter of course, be very likely to appear as disease of the other.

Another anomaly is the appearance of the fistulous opening at some point remote from the seat of disease. Ordinarily, the opening is found directly opposite the end of the root and upon the mucus membrane covering the alveolus; abscesses of the external roots of the compound teeth, and of the oral teeth appearing upon the buccal and labial aspect of the jaw, and those of the palatine roots of the compound teeth upon the palatine side of the jaw.

The anomalous positions noted in the text books may be named as follows:

Fistulous openings have been noted as existing upon the cheek at various points, as far back as the angle of the jaw, the center of the chin, at various points of the palate, remote from the roots of the teeth, even as far back as the point of union of the palatine plates of the superior maxillary bones with the palate bones. (It having been my privilege to treat a case with an opening at the point formed by the union of these four bones, in connection with a diseased lateral incisor.) The opening has also been observed upon the lingual aspect of the mucus membrane covering the under jaw.

Sometimes, especially in case of retarded eruption of the lower third molars, the secretions may pass along the line of the roots upward into the mouth.

In extremely rare cases abscesses have pointed midway between the *pomum Adami* and the sternum, and even as low as the upper border of the clavicle.

Another anomaly is that in which more than one fistulous opening exists in connection with the same tooth. Such a case was treated by me over ten years ago, it being that of an upper molar in which the three roots were diseased, and each had an independent fistulous opening, two upon the buccal and one upon the palatine side, and through each of which fluids were injected by introducing the syringe point into a hole drilled through the crown of the tooth.

Another variation is that in which there is more than one opening on the gum in connection with the same root. Such a case as the last is at present under treatment by me, it being a lower left canine, one opening being one-eighth of an inch above the other.

The two anomalous conditions that are to be reported in this paper will be fully described, and are, so far as it has been possible to determine, entirely unique, the peculiarity in one case consisting in the fact that the fistulous opening was the common point of exit for abscesses at the ends of the roots of two teeth, they being the left superior central and lateral incisors; the other an obscure case of discharge of an abscess into the antrum.

Case 1 was that of a lady about twenty-five years of age. The case was apparently that of an ordinary chronic alveolar abscess connected with an upper left central incisor and having a fistulous opening upon the palate at a point opposite the end of the root. The tooth was quite discolored and was filled with gold upon both proximal faces, and also upon the palatine face at the point usually utilized in entering the pulp chamber for treating such a case.

The fistulous opening was quite large and the discharge of thin, foul-smelling fluid quite profuse.

The lady furnished the following history of the case: The tooth had been filled for a number of years, had remained comfortable for a time, but finally caused the face to swell and was spontaneously vented on the palate. About a year before she applied to me another dentist treated the tooth for a time, filled the canal, pulp chamber and cavity, and pronounced the case cured; the discharge had not ceased at any time, however, and the lady was naturally not satisfied with the result.

Upon learning these facts I advised her to again apply to her dentist and request him to endeavor to complete the cure, at the same time stating to her that in order for me to learn what he already knew it would be necessary for me to take out the filling. This was done because from her description of the case it was thought that the proper course had been followed and that the dentist was fully competent to treat the case successfully. Acting upon my advice the dentist was consulted, and informed her that he could not or would not do anything further in the matter.

I then consented to take charge of the case, and an appointment was given for the purpose of opening the tooth. Upon removal of the filling on the palatine face the chamber and canal were found to be occupied with cotton saturated with carbolic acid. The cotton was discolored and emitted a very unpleasant odor. Tr. iodine, when injected through the tooth, appeared at the orifice of the fistula and caused the desired burning sensation.

Thinking that the nature and condition of the filling material in the root were the cause of the persistence of the discharge, I so informed my patient and predicted a favorable termination of the trouble.

The canal was straightened and enlarged in the usual manner and the tooth treated with creasote. It was dressed twice a week for about one month, when all evidence of secretion of fluid was absent. To make sure of the cure the tooth was kept sealed for two weeks longer, the dressing having been changed four or five times during this period, when an appointment was given and the root filled with gold, and the pulp-chamber and cavity with oxyphosphate of zinc.

The tooth remained quiet for fully three months, when to my mortification the lady returned to report the reappearance of the discharge at the former site of the fistulous opening. Examination realized my worst fears; fluids were being evacuated at this point and no mistake.

The lady was so discouraged that she solicited me to extract the unfortunate tooth and thus put an end to her trouble.

Knowing the root to have been properly filled, I attributed the trouble to the final efforts of Nature to repair the trouble, and injected Tr. iodine through the fistula two or three times, but without causing the cessation of the discharge.

Up to this time the lateral incisor had entirely escaped suspicion, but in examining the teeth in the immediate vicinity a slight opacity was noticed, but was attributed to the presence of a bone filling upon the anterior proximal face.

Although I had never known or heard of a case of two abscesses evacuating at the same point, my suspicion of the lateral was strong enough to cause me to test the vitality of the tooth by sharply striking the crown in a line with the axis, and which caused some pain; cold was applied and no response elicited; a drill was then applied to the palatine face and the enamel pierced, with no pain; the drilling continued into the dentine, and still there was no pain; finally the pulp-chamber was entered and the absence of the nerve verified. The opening was enlarged and Tr. iodine injected, when to my gratification, the well known color of this medicine appeared at the old opening on the palate, and the diagnosis was completed.

Again assuring my patient of a probability of a cure of the trouble, this tooth was treated in a manner similar to that resorted to in case of the adjoining central incisor, and filled in a like manner in about a month after discovery of the trouble.

This was about three months ago, and the lady has been happy ever since.

With the exception of a suspicion of recurrence, caused by a swelling at the point of the former site of the fistulous opening, but which proved to be a slight and transient inflammation of one of the rugae, there has been no further trouble.

Case II was that of a gentleman about sixty years of age, apparently in perfect health, who came under my care for the purpose of having some carious teeth filled.

In addition to caries, phagedenic pericementitis was also present, and had caused the recession of the gum at various points about the teeth. The appointments for the different operations extended over a number of weeks, and toward the latter part of the time he incidentally mentioned the fact that he had been obliged to consult a mutual medical friend on account of a catarrhal affection of the nasal passages.

Knowing that he was under proper care, no attention was paid this disease by me.

Amongst the teeth treated was a right upper second molar, which exhibited much sensitiveness of the dentine on the buccal side, below the termination of the enamel.

To overcome this, strong chloride of zinc was applied several times with benefit, but without completing the cure; finally, the nitrate of silver was applied in full strength (saturated solution), and the sensitiveness in a short time entirely disappeared.

About a fortnight after he had been dismissed he called and stated that his medical attendant had failed in obtaining such action from the remedies he had employed as he had expected, and had directed him to apply to me to have this molar extracted, as he suspected it might be the cause of the catarrhal trouble. On account of my previous experience with this particular tooth, I was, of course, perfectly certain in my own mind that it could have nothing to do with the nasal disease, and objected to carrying out the direction of our medical friend, at the same time explaining to the patient that in order to have the abscess evacuate through the antrum the nerve must have been dead, which the sensitiveness of the dentine, previously treated, would have rendered impossible. However, it was remembered that no inspection of the tooth had been made by me since the sensitiveness of the dentine had been relieved, and it was thought best to be sure the pulp was alive at the present time. Examination with a fine point showed the dentine at the exposed point on the buccal side to be entirely free from sensitiveness, in fact, rather too much so. The scratching with the point was continued higher up under the gum, and no response elicited. The cold and mirror tests were next used, and the pulp discovered to be devitalized.

A fine probe of platinum wire was next passed along the line of the palatine root and found to enter the antrum.

To be perfectly sure of the diagnosis the probe was carried nearly to the floor of the orbit.

You may be sure no time was lost in carrying out the doctor's wishes. Examination after extraction showed the palatine root to be badly discolored, but the buccal roots were only slightly so.

With this newly acquired evidence the case was made out as follows:

The pulp and palatine filament of the nerve had died, causing abscess which had discharged into the antrum, and from thence into the nose, all of which took place at the time the pseudocatarrh was first noticed. The filaments in the buccal roots remained alive as evidenced by the sensitiveness of the dentine in that region.

The medicines used by me to relieve this condition, or possibly the dead pulp, had caused the death of the buccal filaments later on, thus making a diagnosis of alveolar abscess possible.

In this case, as you will observe, there were two peculiar complications, one of which is quite rare:

First. The discharge of the abscess into the antrum.

Second. The death of the pulp and nerve thread in the palatine root so long in advance of those in the buccal roots.

This simple operation (extraction) caused the nasal trouble to disappear at once, the drainage taking place through the socket of the extracted tooth, and the cure was completed in about a week.

This account finishes the history of these, to me, most interesting cases, and which will, it is to be hoped, justify my having selected the title of this paper.

